# Relation of Disaster Exposure With Maternal Characteristics and Obstetric Outcomes: the Tohoku Medical Megabank Project Birth and Three-Generation Cohort Study

**DOI:** 10.2188/jea.JE20210052

**Published:** 2023-03-05

**Authors:** Mami Ishikuro, Taku Obara, Keiko Murakami, Fumihiko Ueno, Aoi Noda, Masahiro Kikuya, Junichi Sugawara, Hirohito Metoki, Shinichi Kuriyama

**Affiliations:** 1 Tohoku Medical Megabank Organization, Tohoku University, Miyagi, Japan; 2 Tohoku University Graduate School of Medicine, Miyagi, Japan; 3 Tohoku University Hospital, Miyagi, Japan; 4 Teikyo University School of Medicine, Tokyo, Japan; 5 Faculty of Medicine, Tohoku Medical and Pharmaceutical University, Miyagi, Japan; 6 International Research Institute of Disaster Science, Tohoku University, Miyagi, Japan

**Keywords:** earthquake, pre-pregnancy body mass index, hypertensive disorders of pregnancy, gestational diabetes mellitus, gestational weeks

## Abstract

**Background:**

The present study analyzed the relation of disaster exposure prior to pregnancy with maternal characteristics and obstetric outcomes.

**Methods:**

The participants were 13,148 pregnant women recruited from 2013 to 2017. The women were classified into three groups by the severity of housing damage caused by the Great East Japan Earthquake of 2011: group A, house was not destroyed/did not live in the disaster area; group B, half/part of the house was destroyed; and group C, house was totally/mostly destroyed. Maternal characteristics, hypertensive disorders of pregnancy (HDP), gestational diabetes mellitus (GDM), and gestational weeks were obtained using questionnaires and medical records. Multiple logistic regression analyses were performed to investigate the relation between disaster exposure and maternal characteristics, HDP, and GDM. A structural equation model was applied to investigate the relation of disaster exposure with HDP and gestational weeks.

**Results:**

The homes of about 11% of the women were totally/mostly destroyed. For groups B and C compared with those in group A, the adjusted ORs for HDP were 1.04 and 1.26 (*P* for trend = 0.01), and for GDM were 0.89 and 1.14 (*P* for trend = 0.9), respectively. Pre-pregnancy body mass index (BMI) mediated 23.2% of the relation between disaster exposure and HDP. Disaster exposure was associated with gestational weeks.

**Conclusion:**

Disaster exposure at least 2.5 years before pregnancy was found to be associated with maternal characteristics and the prevalence of HDP. Pre-pregnancy BMI mediated the relation between disaster exposure and the prevalence of HDP, and gestational weeks were reduced through HDP.

## INTRODUCTION

Catastrophic disasters affect the health and lives of people around the world, and these effects sometimes continue over the long term. A systematic review and meta-analysis of several studies investigating the association between earthquakes and health in high-income countries found that earthquakes increased the risk of myocardial infarction, stroke, and all-cause mortality.^1^ Disasters also affect health-related status, including under/overweight, smoking, alcohol drinking, and psychological distress.^2^^–^^4^ For example, increased body mass index (BMI) has been observed among individuals exposed to famine,^2^ and smoking relapse among those affected by Hurricane Katrina as a result of posttraumatic stress.^3^ These health-related factors could lead to long-term adverse health effects. In particular, people who experience a disaster might incur some additional risks if they become pregnant. Few studies have investigated the associations between disaster exposure, health-related status, and subsequent risk factors during pregnancy.^5^ A previous study investigating the effects of Hurricane Katrina on birth outcomes reported that experience of injury and the presence of continuous physiological distress led to reduce gestational age among pregnant women even at 5–7 years after the disaster.^5^ Therefore, more evidence regarding the long-term effects of a disaster on pregnancy and childbirth needs to be accumulated to help prevent subsequent generations from suffering ongoing adverse health effects.

On March 11, 2011, the Great East Japan Earthquake (GEJE) hit northeast Japan, followed by a devastating tsunami and nuclear disaster. As a result, at least 15,899 lives were lost, and more than 120,000 houses and buildings were totally destroyed.^6^ After the disaster, an increase in maternal health problems was observed.^7^^,^^8^ For instance, emergency obstetric transport increased 1.4 times compared with before the disaster,^7^ and pregnant women who had to attend different medical facilities because of the GEJE delivered earlier.^8^ However, to our knowledge, no study has investigated whether experiencing such a massive earthquake and tsunami in the preconception period affects long-term maternal health-related status and obstetric outcomes.

This study investigated maternal characteristics and the associations between maternal disaster exposure before pregnancy and obstetric outcomes typically affected by maternal characteristics from at least 2.5 years after the GEJE.

## METHODS

### Design

The present study is part of the Tohoku Medical Megabank Birth and Three-Generation Cohort Study (TMM BirThree Cohort Study). The purpose of the TMM BirThree Cohort Study is to investigate the effects of the disaster on people’s health and identify their needs for healthcare services, and to establish precision medicine/healthcare for providing better care for people living in the disaster area and all over Japan. The protocol of the TMM BirThree Cohort Study was approved by the Tohoku Medical Megabank Organization internal review board (No. 2013-1-103-1). For the TMM BirThree Cohort Study, written informed consent was obtained from the participants. The detailed design of the study has been described elsewhere.^9^^,^^10^

### Participants

Overall, 22,493 pregnant women who had been recruited to participate in the TMM BirThree Cohort Study in Miyagi Prefecture from 2013 to 2017 were targeted in the present study. Women who withdrew consent (*n* = 347), did not answer the questionnaire (*n* = 8,895), or did not answer the question item about if their houses had been destroyed in the GEJE (*n* = 103), which was a proxy to measure their disaster exposure in this study, were excluded. Therefore, 13,148 women (58.5%) were finally eligible for analysis. The basic characteristics of eligible and not eligible people (except for withdrawals) are shown in eTable 1.

### Materials

The severity of housing damage was obtained through a questionnaire at 12 months after childbirth based on the following categories: 1) totally destroyed; 2) mostly destroyed; 3) half-destroyed; 4) partly destroyed; 5) not destroyed; and 6) did not live in the disaster-affected area. Disaster exposure was then categorized by the severity of housing damage into three groups: group A, house was not destroyed/did not live in the disaster-affected area; group B, half/part of the house destroyed; and group C, house totally/mostly destroyed. Maternal characteristics, including pre-pregnancy height, pre-pregnancy weight, smoking status, scores on the Kessler Psychological Distress Scale-6 (K6), and status regarding living with parents, were obtained through a questionnaire in the first trimester, and household income was obtained from a questionnaire in the second trimester. Pre-pregnancy BMI was estimated using the pre-pregnancy height and weight obtained through the questionnaires. Parity, multiple pregnancies, blood pressure (BP), and urological test results during pregnancy was extracted from medical records by genome medical research coordinators hired by the Tohoku Medical Megabank Organization. Age at conception was calculated based on the estimated date of confinement extracted from medical records and the birth dates of the pregnant women. Regarding obstetric diseases, the study focused on hypertensive disorders of pregnancy (HDP) and gestational diabetes mellitus (GDM), the risks of which are increased according to health-related status. HDP were identified by BP level and urological test results from medical records in accordance with the criteria of the American College of Obstetricians and Gynecologists.^11^ GDM was identified based on the results of an antenatal oral glucose tolerance test from medical records in accordance with the criteria of the International Association of Diabetes and Pregnancy Study Groups.^12^ Birth weight and gestational weeks of newborns were extracted from medical records.

### Statistical analyses

Maternal characteristics were compared between the three groups using the chi-squared test. Multiple logistic regression analyses were performed to investigate the associations between disaster exposure and pre-pregnancy BMI, smoking in the first trimester, K6 score ≥13 (the cutoff value for psychological distress in the first trimester), household income <4 million Japanese yen (JPY)/year, and status of living with parents in the first trimester. *P* value for trend was calculated for each variable.

To compare the proportions of HDP and GDM between the three groups, the chi-squared test and Cochran–Armitage test were used. Multiple logistic regression analyses were performed to investigate the relation between disaster exposure, HDP, and GDM adjusted for age at conception, pre-pregnancy BMI, history of HDP/GDM, and family history of hypertension/diabetes mellitus.^13^^–^^16^ Participation year was also adjusted in the model because the conception period varied. The status of living with parents in the first trimester and its association with disaster exposure was identified, and was also adjusted in the model. HDP were additionally adjusted for multiple pregnancies and smoking status in the first trimester, which are also possible risk factors for HDP according to a previous study.^15^ For birth weight and gestational weeks, analysis of variance were performed between three groups, and for low birth weight (LBW) (<2,500 g), and preterm birth (<37 gestational weeks) between the three groups, the chi-squared test and Cochran–Armitage test were used.

As sub analyses, a structural equation model was applied to examine whether pre-pregnancy BMI might be a mediator between disaster exposure and the prevalence of HDP because pre-pregnancy BMI was associated with both disaster exposure and the prevalence of HDP in the multiple logistic regression analyses. In the model, pre-pregnancy BMI was used as a continuous variable because significant linear trends were found in the multiple logistic regression analyses. The model was adjusted for age at conception, family history of hypertension, multiple pregnancies, and nulliparous or multiparous women with a history of HDP. Those variables were examined in regard to their association with the prevalence of HDP in the multiple logistic regression analysis. Nulliparous and multiparous women with a history of HDP were categorized together and compared with multiparous women without a history of HDP because the model only allowed dichotomous variables for categorical data. No association was observed between maternal disaster exposure and GDM on univariate nor multivariate analyses; therefore, the study did not apply a structural equation model to explain the direct and indirect effects between maternal disaster exposure and GDM. The study also applied a structural equation model to investigate whether pre-pregnancy BMI and HDP might mediate between maternal disaster exposure and gestational weeks. Gestational weeks were applied as a continuous variable, as a relation with disaster exposure was observed on bivariate analysis. The acceptability of both structural equation models was assessed as follows: the smallest chi-square value with *P* > 0.05, comparative fit index >0.95, and root mean square error of approximation <0.05. All statistical analyses were performed using SAS (version 9.4; SAS Institute Inc., Cary, NC, USA).

## RESULTS

Overall, the mean/median maternal age at conception was 31.3/31 (standard deviation, 4.9; interquartile range, 28–35) years, and 6,326 (48.1%) women were nulliparous. The homes of about 11% of the women were totally/mostly destroyed.

### Disaster exposure and maternal characteristics from at least 2.5 years after the disaster

The proportion of women with a pre-pregnancy BMI ≥25 kg/m^2^ was higher in group C than in groups A and B (Table 1). The proportion of women who continued to smoke during the first trimester was also higher in group C than in groups A and B. The proportion of women who had a K6 score ≥13 in the first trimester tended to be higher in group C than in groups A and B; however, the use of sleeping pills was not significantly different between the three groups (*P* = 0.1). The proportion of women with a household income <4 million JPY/year was higher in group C than in groups A and B. More women in group C lived with their parents compared with the other two groups.

**Table 1.** Participants’ basic characteristics

(*n* = 13,148)							

	Group A: House was not destroyed/did not live in the disaster-affected area (*n* = 6,838)		Group B: House half-/partly destroyed (*n* = 4,878)		Group C: House totally/mostly destroyed (*n* = 1,434)		*P*
		
	*n*	%	*n*	%	*n*	%	
Age, ≥35 years	1,757	25.8	1,439	29.6	358	25.0	<0.0001
Primiparous	3,424	50.2	2,239	46.0	663	46.4	<0.0001
Pre-pregnancy BMI							0.04
<18.5 kg/m^2^	1,113	16.3	760	15.6	215	15.0	
≥25 kg/m^2^	706	10.3	552	11.3	186	13.0	
Fertility treatments (Yes)	815	12.0	534	11.1	142	10.0	0.047
Multiple pregnancy (Yes)	88	1.9	76	2.3	13	1.4	0.1
Smoking during the first trimester							<0.0001
Current smoker	114	1.7	89	1.8	35	2.5	
Stopped after becoming pregnant	735	10.9	590	12.2	224	15.8	
Stopped before pregnancy	1,525	22.5	1,226	25.4	350	24.6	
Never	4,396	64.9	2,930	60.6	812	57.1	
Drinking during the first trimester							0.0006
Current drinker	1,406	20.8	1,034	21.4	235	16.5	
Stopped before or during pregnancy	2,321	34.3	1,624	33.6	479	33.7	
Never	3,047	45.0	2,181	45.1	707	49.8	
Strong activity once or more per week before pregnancy (Yes)	1,067	15.8	787	16.3	240	16.9	0.5
Sleeping pills							0.1
Never use	6,737	99.3	4,807	99.3	1,409	98.9	
Less than once per week	13	0.2	6	0.1	1	0.07	
Once or more per week	34	0.5	29	0.6	15	1.1	
K6 score, ≥13	360	5.3	276	5.7	97	6.8	0.08
Household income (Japanese yen)							<0.0001
<4 million	2,119	32.9	1,627	35.6	591	43.8	
≥4 million, <8 million	3,478	53.9	2,328	50.9	626	46.4	
≥8 million	854	13.2	618	13.5	133	9.9	
Living with Partner	6,274	91.8	4,464	91.5	1,292	90.1	0.1
Living with Maternal parents	537	7.9	544	11.2	182	12.7	<0.0001
Living with Paternal parents	866	12.7	751	15.4	249	17.4	<0.0001
Year of participation							0.01
2013	327	4.8	254	5.2	52	3.6	
2014	2,068	30.3	1,606	32.9	475	33.1	
2015	2,592	37.9	1,780	36.5	514	35.8	
2016	1,841	26.9	1,232	25.3	392	27.3	
2017	8	0.1	6	0.1	1	0.07	

BMI, body mass index; K6, Kessler 6 Psychological Distress Scale.

**Figure 1.**
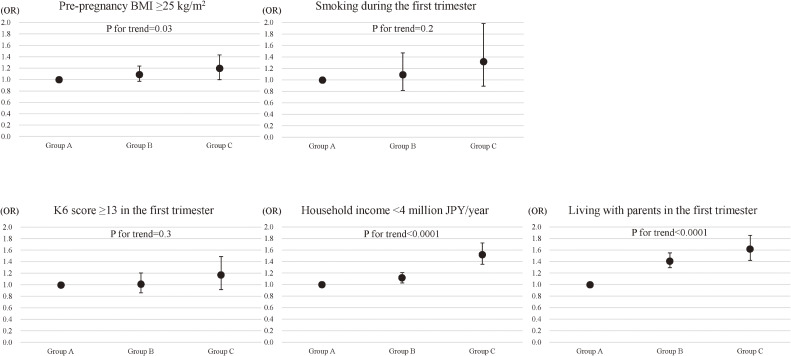
Associations between disaster exposure and maternal characteristics. The association between disaster exposure and each maternal characteristic, such as pre-pregnancy body mass index (BMI) ≥25 kg/m^2^, smoking during the first trimester, score ≥5 on the Kessler Psychological Distress Scale-6 (K6) Psychological Distress Scale in the first trimester, household income <4 million JPY/year, and living with the parents in the first trimester as identified by multiple logistic regression analyses are shown. Multiple logistic regression analysis for the association between disaster exposure and pre-pregnancy BMI ≥25 kg/m^2^ was adjusted for smoking during the first trimester, K6 score ≥13 in the first trimester, household income <4 million JPY/year, and living with the parents in the first trimester. Smoking during the first trimester was adjusted for pre-pregnancy BMI ≥25 kg/m^2^, K6 score ≥13 in the first trimester, household income <4 million JPY/year, and living with the parents in the first trimester. K6 score ≥13 in the first trimester was adjusted for pre-pregnancy BMI ≥25 kg/m^2^, smoking during the first trimester, household income <4 million JPY/year, and living with the parents in the first trimester. Household income <4 million JPY/year was adjusted for pre-pregnancy BMI ≥25 kg/m^2^, smoking during the first trimester, K6 score ≥13 in the first trimester, and living with the parents in the first trimester. Living with the parents in the first trimester was adjusted for pre-pregnancy BMI ≥25 kg/m^2^, smoking during the first trimester, K6 score ≥13 in the first trimester, and household income <4 million JPY/year. Odds ratios and 95% confidence intervals were calculated for groups B and C, with group A as the reference value. *P* for trends of disaster exposure are shown for each maternal characteristic.

On multivariate analyses, the adjusted odds ratios (ORs) and 95% confidence intervals (CIs) for pre-pregnancy BMI ≥25 kg/m^2^ were 1.09 (95% CI, 0.97–1.24) and 1.20 (95% CI, 0.999–1.43) in groups B and C, respectively, compared with those in group A (*P* for trend = 0.03) even though analyses were adjusted for other variables, such as smoking during the first trimester, K6 score ≥13 in the first trimester, household income <4 million JPY/year, and living with either/both paternal and/or maternal parents in the first trimester (Figure 1). A K6 score ≥13 in the first trimester did not show a positive association with BMI ≥25 kg/m^2^ (OR 1.05; 95% CI, 0.83–1.33) (data not shown in Figure 1). The adjusted ORs for smoking in the first trimester were 1.10 (95% CI, 0.82–1.47) and 1.33 (95% CI, 0.89–1.98) in groups B and C, respectively, compared with those in group A (*P* for trend = 0.2). The adjusted ORs for K6 score ≥13 in the first trimester were 1.02 (95% CI, 0.86–1.20) and 1.17 (95% CI, 0.92–1.49) in groups B and C, respectively, compared with those in group A (*P* for trend = 0.3). Positive associations were found between the severity of housing damage and both household income <4 million JPY/year as well as between the severity of housing damage and living with parents in the first trimester (*P* for trend <0.0001 for each).

### Disaster exposure and obstetric outcomes

The prevalence of HDP tended to be higher with more severe housing damage (10.3%, 10.9%, and 12.8% in groups A, B, and C, respectively: *P* for trend = 0.01) (Table 2). The prevalence of GDM was higher in group C (2.9%) than in groups A and B (2.5% and 2.4%, respectively); however, no trends in the prevalence were found among the three groups. Regarding newborns, the prevalence of neither LBW nor preterm birth differed in association with maternal disaster exposure before participating in the TMM BirThree Cohort Study, while gestational weeks as a continuous variable was lower in group C than in group A. On multiple logistic regression analysis, the adjusted ORs for HDP and GDM were 1.02 (95% CI, 0.91–1.16) and 1.26 (95% CI, 1.05–1.51; *P* for trend = 0.02; Table 3A) and 0.89 (95% CI, 0.70–1.14) and 1.14 (95% CI, 0.81–1.61; *P* for trend = 0.9; Table 3B) in groups B and C compared with those in group A, respectively.

**Table 2.** Univariate analysis of the groups classified by degree of housing damage

(*n* = 13,148)								

Variables	Group A: House was not destroyed/did not live in the disaster-affected area (*n* = 6,838)		Group B: House half-/partly destroyed (*n* = 4,878)		Group C: House totally/mostly destroyed (*n* = 1,434)		*P*	*P* for trend
		
	*n*/means	%/SD	*n*/means	%/SD	*n*/means	%/SD		
Maternal outcomes								
Hypertensive disorders of pregnancy	707	10.3	531	10.9	184	12.8	0.02	0.01
Gestational diabetes mellitus	173	2.5	116	2.4	42	2.9	0.5	0.7

Child outcomes								
Newborn birth weight, g	3,013.6	424.3	3,015.6	446.8	3,009.4	474.9	0.9	0.9
Newborn with low birth weight (<2,500 g)	632	9.3	459	9.4	145	10.1	0.6	0.4
Gestational weeks	38.7	1.7	38.7	1.9	38.6	2.1	0.004	0.001
Preterm birth (<37 gestational weeks)	393	5.8	321	6.6	90	6.3	0.2	0.1

SD, standard deviation.

**Table 3A.** Multiple logistic regression analysis of hypertensive disorders of pregnancy (HDP)

	OR	95% CI	*P* for trend
House destroyed (ref = House was not destroyed/did not live in the disaster-affected area)			0.02
House half-/partly destroyed	1.02	0.91 – 1.16	
House totally/mostly destroyed	1.26	1.05 – 1.51	
Age at conception, ≥35 years (ref <35 years)	1.27	1.13 – 1.44	0.025
Pre-pregnancy BMI (kg/m^2^) (ref ≥18.5 and <25)			<0.0001
<18.5	0.94	0.79 – 1.11	
≥25	3.46	3.01 – 3.98	
Smoking status (ref = Nonsmoker)			0.05
Past smoker	1.16	1.03 – 1.31	
Current smoker	0.98	0.64 – 1.50	
Year joining the study (ref = Year of 2013)			0.2
Year of 2014	1.11	0.83 – 1.48	
Year of 2015	1.08	0.81 – 1.45	
Year of 2016	1.12	0.83 – 1.50	
Year of 2017	0.68	0.09 – 5.40	
History of HDP (ref = Nulliparas)			0.002
Multiparas without a history	0.53	0.47 – 0.60	
Multiparas with a history	2.59	2.01 – 3.34	
Multiple pregnancies (ref = Singleton)	1.53	1.01 – 2.33	0.02
Family history of hypertension	1.49	1.33 – 1.67	<0.0001
Living with maternal parents	1.01	0.83 – 1.22	0.7
Living with paternal parents	0.95	0.81 – 1.13	0.2

BMI, body mass index; CI, confidence interval; OR, odds ratio.

**Table 3B.** Multiple logistic regression analysis of gestational diabetes mellitus (GDM)

	OR	95% CI	*P* for trend
House destroyed (ref = House was not destroyed/did not live in the disaster-affected area)			0.9
House half-/partly destroyed	0.89	0.70 – 1.14	
House totally/mostly destroyed	1.14	0.81 – 1.61	
Age at conception, ≥35 years (ref <35 years)	1.72	1.37 – 2.16	<0.0001
Pre-pregnancy BMI (kg/m^2^) (ref ≥18.5 and <25)			<0.0001
<18.5	0.84	0.59 – 1.20	
≥25	2.57	1.97 – 3.35	
Year joining the study (ref = Year of 2013)			0.003
Year of 2014	0.84	0.48 – 1.47	
Year of 2015	1.29	0.75 – 2.21	
Year of 2016	1.38	0.80 – 2.40	
Year of 2017	N.A.	N.A. N.A.	
History of GDM (ref = Nulliparous women)			0.02
Multiparas without a history	0.96	0.76 – 1.20	
Multiparas with a history	10.88	6.23 – 19.02	
Family history of diabetes mellitus	1.41	1.06 – 1.87	0.004
Living with maternal parents	1.12	0.78 – 1.60	0.5
Living with paternal parents	0.82	0.59 – 1.15	0.3

BMI, body mass index; CI, confidence interval; GDM, gestational diabetes mellitus; OR, odds ratio.

### Mediation analyses for the relation between disaster exposure and HDP

**Figure 2.**
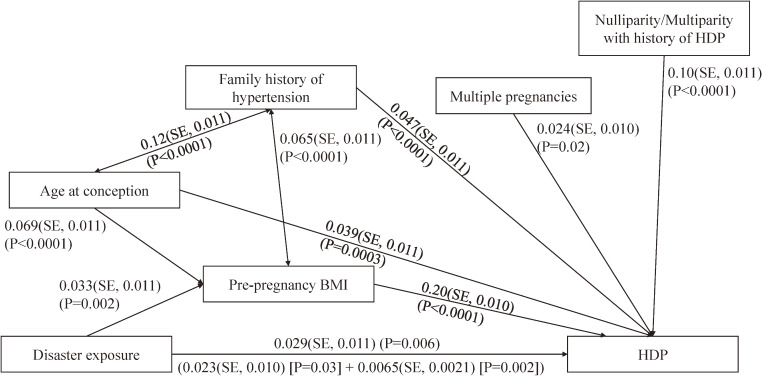
Direct and indirect effects of disaster exposure and hypertensive disorders of pregnancy (HDP). A structural equation model was used to calculate the standardized direct and indirect causal effects between disaster exposure and HDP. Pre-pregnancy body mass index (BMI) was hypothesized as a mediator for both relations between disaster exposure and HDP.

Although the standardized total effect for the relation between disaster exposure and HDP was small (0.029; standard error (SE), 0.011, *P* = 0.006), disaster exposure was identified as a direct factor for HDP (direct effect: 0.023; SE, 0.011, *P* = 0.03; indirect effect: 0.0065; SE, 0.0021, *P* = 0.002; Figure 2). The model fit as a chi-square statistic was 0.16 (*P* = 0.9; comparative fit index = 1.0; and root mean square error of approximation = 0). Pre-pregnancy BMI ≥25 kg/m^2^ mediated the causal association between disaster exposure and HDP.

### Possible pathways from disaster exposure to gestational weeks

**Figure 3.**
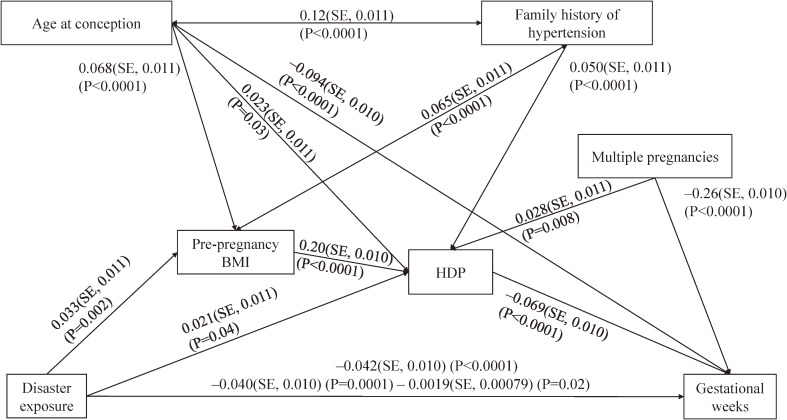
Pre-pregnancy body mass index (BMI) and hypertensive disorders of pregnancy (HDP) occurred after the disaster as possible risk factors for gestational weeks. The standardized results regarding the regression coefficient, standard error, and *P* values are shown.

In the structural equation model for gestational weeks, maternal disaster exposure was negatively associated with gestational weeks (Figure 3). The model fit as a chi-square statistic was 2.26 (*P* = 0.5; comparative fit index = 1.0; and root mean square error of approximation = 0). Gestational weeks was negatively associated with HDP, but not significantly directly associated with pre-pregnancy BMI.

## DISCUSSION

In this study, an association was observed of having one’s house destroyed by the GEJE with maternal characteristics and the prevalence of obstetric diseases more than 2.5 years after the disaster. The launch of the TMM BirThree Cohort Study was in December 2013, and the last birth was in June 2017. The results of this study indicate that the disaster may have affected subsequent pregnancy, even after such a long time.

The proportion of women classified as overweight (BMI ≥25 kg/m^2^) at pre-pregnancy was higher in the housing damage groups B and C than in group A, who did not experience housing damage during the GEJE. In the multiple logistic regression analysis between disaster exposure and overweight at pre-pregnancy, which was adjusted for other maternal characteristics, a tendency for the severity of housing damage to be positively associated with overweight at pre-pregnancy was observed, suggesting that the prevalence of overweight might increase after a disaster. Posttraumatic stress is often reported as being a factor for overweight after a disaster.^17^ We previously observed that psychological distress in women during pregnancy and the postpartum period increased after the GEJE.^18^^–^^20^ In this study, although the proportion of K6 scores ≥13 tended to be higher in group C than in groups A and B, no association was found between K6 scores ≥13 in the first trimester and either severity of housing damage or overweight at pre-pregnancy. This contradiction between previous studies and this study might be explained by differences in the recruitment period, since the range of years was between 2013 and 2017. In addition, the proportion of psychological distress was continuously higher in Miyagi Prefecture during 2011–2013 than that of other areas in 2011.^20^ Therefore, it is possible that not psychological distress but other factors would interact with overweight. Another study reported decreased physical activity and weight gain among over 55 year-olds after the GEJE,^21^ and a systematic review reported that experiencing famine increased the risk of overweight and obesity, especially in individuals aged less than 50 years and in females.^2^ Although the participants in this study were younger, these health behaviors may be associated with overweight at pre-pregnancy.

The proportion of pregnant women who continued to smoke during the first trimester was higher among those who experienced housing damage than among those who did not. However, smoking during the first trimester did not tend to increase according to the severity of housing damage. In Fukushima Prefecture, which is adjacent to our study area and had also been extensively damaged by the tsunami after the GEJE, the proportion of smokers decreased after compared with before the disaster.^22^ However, starting smoking after the disaster was significantly associated with experiencing the tsunami and housing damage.^22^ In particular, 20–49-year-olds had a higher smoking rate than those aged more than 50 years in a previous study.^22^ As our study participants included younger generations, an association between disaster exposure and smoking status might have been observed. Other associations between disaster exposure and maternal characteristics, such as a lower household income and a higher proportion of living with parents, could be partly explained by unemployment or evacuation due to the disaster.

In previous studies, age, BMI, parity, history of HDP, and family history of hypertension were found to be risk factors for HDP.^13^^–^^15^ Regarding the GEJE, women who lived in the disaster-affected area of Fukushima Prefecture during the third trimester of pregnancy were found to have a higher prevalence of HDP.^23^ That previous study divided the disaster-affected area based on the location of the nuclear power plant and observed a relation between experiencing the tsunami or an evacuation and the prevalence of HDP by living area. The present study classified pregnant women by their experience of housing damage as a proxy of disaster exposure, regardless of whether they lived in a coastal area. Therefore, the present study might have assessed more general disaster experience-based exposure, not only tsunami or evacuation experience, but also housing collapse, landslide, or fire damage. In addition, our study considered women who experienced a disaster before becoming pregnant. The results of a mediation analysis indicated that disaster exposure was a possible risk factor for HDP mediated by pre-pregnancy BMI. In the aftermaths of both the Great Hanshin Earthquake and the GEJE, increases in BP were observed.^24^^,^^25^ The direct effect of disaster experience on HDP can be partially explained by sympathetic activation because of the stress caused by the disaster and environmental change.^24^ In addition, as about 2.5 years had passed since the disaster, pre-pregnancy BMI, which was associated with disaster experience among pregnant women, might have mediated the pathway from disaster experience to HDP.

The present study also investigated the relation between disaster exposure and GDM; however, no association was observed. To our knowledge, no previous study has found an association between disaster exposure and GDM. However, BMI has been reported to be a significant risk factor for maternal obstetric outcomes, such as HDP and GDM, as well as for hypertension or cardiovascular diseases in later life.^13^^,^^14^^,^^26^ Therefore, a more individual approach is needed to help prevent the occurrence of such diseases in overweight women.

In this study, maternal disaster experience was found to be associated with shortened gestational weeks, but not with preterm birth or LBW. According to a systematic review of the effects of disasters on maternal and child health, no types of disasters appeared to increase the prevalence of preterm birth or reduce gestational duration, but some did increase LBW.^27^ One study reported that the OR of preterm birth among pregnant women living in a coastal area, who were supposed to be affected by the GEJE more than women living in an inland area, was 0.85, with no statistical significance.^28^ Another study using vital statistics of Japan reported that although the birthweight of males born to mothers who experienced the GEJE during 28 to 36 gestational weeks in Miyagi, Iwate, and Fukushima Prefectures was lower than that of males born in 2010, the difference was only 16 g; thus, the effect of the GEJE on perinatal outcomes might have been small.^29^ Our results are inconsistent with those reported in previous studies. The results in more recent studies than the prior systematic review^27^ are still controversial. The project Ice Storm found that women who experienced the ice storm during early to mid pregnancy delivered their babies earlier or with lower birthweight compared to women who experienced the ice storm during the third trimester and pre-pregnancy exposure.^30^ Another study investigated the effect of Hurricane Katrina on pregnancy at 5–7 years after the disaster,^5^ and it found that any disaster-related experience, such as housing damage or bodily injury, increased the risk of preterm birth. In fact, the structural equation model used in the present study found that HDP mediated by pre-pregnancy BMI were negatively associated with gestational weeks, though the proportion of preterm birth was not different by housing damage. Furthermore, another study noted the potential effects of a disaster on subsequent generations. Handel et al^31^ noted that an increased prevalence of LBW, which is a risk factor for metabolic diseases, was observed after several natural disasters. They also mentioned a Swedish study revealing that food availability among grandparents affected the mortality of grandchildren. HDP are an established risk factor for LBW and preterm birth, as is GDM for preterm birth. The present study, therefore, provides new insight into the relation between large earthquakes followed by a tsunami and long-term maternal outcomes. Whether such catastrophes affect multiple generations, especially in terms of HDP and GDM, needs to be investigated in a future study.

This study have limitations. First, the data on housing damage were limited because the present study used a questionnaire survey at 12 months after childbirth for the data collection; therefore, it is possible that women who had a severe health condition or women who were too busy were not involved in our analysis. The TMM BirThree Cohort Study did not ask pregnant women such sensitive questions during pregnancy to avoid having any effects on maternal or fetal health. As shown in eTable 1, eligible participants were less overweight, had fewer LBW babies, and were less likely to present with preterm birth. Although the proportion of HDP and GDM among eligible participants did not differ from non-eligible participants, we cannot exclude the possibility that the effects of the disaster were underestimated. In addition, assessment of housing damage using questionnaires might have recall bias. However, the Great East Japan Earthquake is one of the biggest natural disaster in history and unforgettable, so the bias might be small. Finally, the present study could not compare characteristics of pregnant women before and after the disaster; therefore, it is possible that differences reflect their characteristics having before the disaster. However, the present study compared pregnant women based on their housing damage by the disaster in all Miyagi Prefecture, so the severity of the disaster reflected our results more than their characteristics having before the disaster.

In conclusion, in this study, disaster exposure at least 2.5 years before pregnancy was found to be associated with maternal characteristics and the prevalence of HDP. Furthermore, pre-pregnancy BMI mediated the relation between disaster exposure and the prevalence of HDP, and gestational weeks were reduced through HDP.

## SUPPLEMENTARY MATERIAL

eTable 1. Comparison of basic characteristics and outcomes between eligible and not eligible participants for the analysis
