# Supplementary material for: Relation of Disaster Exposure With Maternal Characteristics and Obstetric Outcomes: the Tohoku Medical Megabank Project Birth and Three-Generation Cohort Study
Source: J Epidemiol. 2023 Mar 5;33(3):127–35. doi: 10.2188/jea.JE20210052 (PMC9909171; doi:10.2188/jea.JE20210052)
Supplement: Supplementary file 1 [file je-33-127-s001.pdf]

eTable 1. Comparison of basic characteristics and outcomes between eligible and not eligible participants for the analysis

	Not eligible n=8,998		Eligible n=13,148		*P*
	n	%/SD	n	%/SD	
Age, ≥35 years	1,984	22.3	3,554	27.1	<0.0001
Primiparous	4,427	49.4	6,326	48.2	0.1
Pre-pregnancy BMI					<0.0001
<18.5 kg/m^2	2,177	24.2	2,088	15.9	
≥25 kg/m^2	1,031	11.5	1,444	11.0	
Fertility treatments (Yes)	711	8.7	1,491	11.4	<0.0001
Multiple pregnancy (Yes)	128	2.1	177	2.0	0.8
Smoking during the first trimester					<0.0001
Current smoker	300	3.7	238	1.8	
Stopped after becoming pregnant	1,508	18.5	1,549	11.9	
Stopped before pregnancy	1,835	22.5	3,101	23.8	
Never	4,522	55.4	8,138	62.5	
Drinking during the first trimester					0.001
Current drinker	1,522	18.6	2,675	20.5	
Stopped before or during pregnancy	2,912	35.6	4,424	33.9	
Never	3,743	45.8	5,935	45.5	
Strong activity once or more per week before pregnancy (Yes)	1,350	16.5	2,094	16.1	0.4

Sleeping pills					0.2
Never use	8,109	99.0	12,953	99.3	
Less than once per week	21	0.3	20	0.2	
Once or more per week	58	0.7	78	0.6	
K6 score, ≥13	501	6.2	733	5.6	0.1
Household income (Japanese yen)					<0.0001
<4 million	2,883	39.3	4,337	35.1	
≥4 million, <8 million	3,618	49.4	6,432	52.0	
≥8 million	828	11.3	1,605	13.0	
Living with Partner	7,325	81.4	12,030	91.5	<0.0001
Living with Maternal parents	1,034	11.5	1,263	9.6	<0.0001
Living with Paternal parents	1,213	13.5	1,866	14.2	0.1
Family history of hypertension	28	0.3	4,913	37.4	<0.0001
Family history of diabetes mellitus	8	0.1	1,666	12.7	<0.0001
Year of participation					<0.0001
2013	255	2.8	633	4.8	
2014	2,476	27.5	4,149	31.6	
2015	3,642	40.5	4,886	37.2	
2016	2,617	29.1	3,465	26.4	
2017	8	0.1	15	0.1	
Maternal outcomes					
Hypertensive disorders of pregnancy	1,010	11.2	1,422	10.8	0.3
Gestational diabetes mellitus	261	2.9	331	2.5	0.08

Child outcomes					
Newborn birth weight, g	2,972.5	559.8	3,013.9	438.5	<0.0001
Newborn with low birth weight (<2,500 g)	1,309	14.6	1,236	9.4	<0.0001
Gestational weeks	38.3	3.2	38.7	1.8	<0.0001
Preterm birth (<37 gestational weeks)	1,055	11.7	804	6.1	<0.0001

BMI, body mass index; K6, Kessler 6 Psychological Distress Scale; SD, standard deviation.

The chi-squared test for categorical variables and the Student's t test for continuous variables were used for the comparison.
